# Development and validation of a disability risk prediction model in readmitted recurrent stroke patients

**DOI:** 10.3389/fneur.2026.1890175

**Published:** 2026-09-16

**Authors:** Linghui Kong, Peng Jiang, Jing Wang, Huan Qiao, Tao Feng

**Affiliations:** Linfen Central Hospital, Linfen, China

**Keywords:** disability, nomogram, recurrent readmission, risk prediction, stroke

## Abstract

**Objective:**

To analyze the risk factors of disability in readmitted patients with recurrent stroke and construct a nomogram model, so as to provide references for clinical medical staff to formulate targeted intervention measures for stroke patients.

**Methods:**

From September 2023 to February 2025, a total of 415 readmitted patients with recurrent stroke were enrolled from the neurology departments of tertiary hospitals in Liaoning and Shanxi Provinces by convenience sampling method. Chi-square test and *t*-test were used for univariate analysis, and binary logistic regression analysis was performed to establish the risk prediction model. A nomogram was plotted to visualize the risk, and the model was validated via the area under the ROC curve and calibration curve.

**Results:**

The detection rate of disability among readmitted patients with recurrent stroke was 60%. Complicated hypertension, depressive symptoms, cognitive impairment (CI), occasional or no physical exercise were independent risk factors for disability (*p* < 0.05), while social support and self-efficacy were protective factors (*p* < 0.05). The AUC value was 0.993. The calibration curve was well fitted with the ideal curve, and the clinical decision curve confirmed favorable clinical application value of the model.

**Conclusion:**

The constructed nomogram model presents satisfactory predictive efficiency. It can assist clinical staff in screening disability risks, and carry out targeted intervention and rehabilitation management according to relevant risk factors, so as to reduce the incidence of disability in readmitted patients with recurrent stroke.

## Introduction

Stroke, also known as cerebrovascular accident (CVA), is a cerebrovascular disease caused by sudden rupture or occlusion of intracranial blood vessels. It leads to abnormal cerebral blood supply and subsequent organic damage to brain tissues, mainly categorized into ischemic stroke (cerebral infarction) and hemorrhagic stroke (1). Currently, stroke ranks first among diseases with the highest mortality and disability rates in China, and its incidence keeps rising year by year. Patients commonly suffer from varying degrees of neurological deficits involving speech, motor and sensory functions. Coupled with high recurrence and readmission rates, stroke has become a major concern in clinical diagnosis and treatment (2).

The China National Stroke Registry (CNSR) found that 60% of patients with first-ever acute stroke face recurrence risks (3), with the highest risk occurring within 6 months after initial onset (4–6). Relevant cohort studies have demonstrated that the 30-day readmission rate reaches 12.4% among adult patients with ischemic stroke after discharge (7). Elderly patients present a higher short-term readmission rate, which aggravates disability and markedly increases mortality risk. Accordingly, stroke readmission rate has been recognized domestically and internationally as a core indicator for evaluating therapeutic efficacy and monitoring disease progression (8). Effective control of this indicator has become a key priority in clinical management and treatment across medical institutions.

Disability refers to the loss or limitation of fundamental daily living abilities, serving as a vital indicator for individual health assessment (9). Stroke is identified as one of the leading causes of physical disability (10). Most survivors suffer from hemiplegia, numbness, cognitive impairment, aphasia, dysphagia and depression of varying severity, resulting in impairments in physical function, occupational activity, communication and social participation, and severely impairing quality of life (11, 12). Compared with first-ever stroke patients, those with recurrent stroke and readmission have notably higher disability and mortality rates (13). Increased recurrence times correspond to declined activities of daily living scores and aggravated disability (14). Mohan et al. (15) reported the 5-year cumulative stroke recurrence risk was 26.4%, which rose to 39.2% in the 10-year follow-up (16). High recurrence tends to trigger or worsen disability, further deteriorating life quality and even causing death (17). Relevant research indicates the disability rate of readmitted patients with recurrent ischemic stroke reaches 57.2% (18). In our cohort, the disability rate of recurrent stroke patients rehospitalized for stroke relapse further climbed to 60.0%, demonstrating that disability has become an extremely prominent clinical threat to physical function and life quality among this specific patient subgroup.

In recent years, multiple prognostic prediction models targeting stroke-related disability have been developed at home and abroad, yet systematic sorting and narrative review focusing specifically on readmitted recurrent stroke populations remain insufficient, and core research gaps in this subdivision have not been clearly defined in existing literature. Current stroke disability prediction research suffers from four prominent deficiencies that restrict clinical translational value, as summarized below:

First, most existing models mix first-ever stroke patients and recurrent stroke patients without stratified subgroup analysis, ignoring the distinct heterogeneity of functional prognosis across different stroke populations (19). Four stroke subgroups with completely different clinical risk profiles can be distinguished: first-ever stroke inpatients, stable recurrent stroke outpatients, mixed sample stroke cohorts, and recurrent stroke patients readmitted due to disease deterioration. For readmitted recurrent stroke patients, repeated cerebral vascular injury, cumulative chronic comorbidities, accumulated psychological stress, and interrupted rehabilitation training jointly construct a unique disability risk spectrum that cannot be reflected in models built on mixed or non-readmitted samples (20). Models derived from general stroke populations overestimate or underestimate disability risk when applied to rehospitalized recurrent stroke patients, leading to poor predictive validity for this high-risk subgroup.

Second, the vast majority of current predictive tools target general stroke inpatients or community-dwelling stroke survivors, while few models specifically center on patients with recurrent stroke complicated by readmission events (21). Unplanned readmission itself is a powerful marker of poor long-term functional prognosis, representing unstable vascular control, aggravated neurological damage, and failed secondary prevention. Existing disability prediction models rarely incorporate readmission status as a core stratification factor, and thus fail to capture the compound adverse effects of repeated stroke attacks and rehospitalization on daily living ability decline. To date, no individualized quantitative prediction model exclusively for readmitted recurrent stroke patients has been reported in domestic and international research, forming an obvious clinical research blank.

Third, mainstream stroke disability prediction models have prominent methodological limitations inconsistent with TRIPOD reporting standards for prognostic models (22). (1) Lack of visualized nomogram tools: Most studies only output complex logistic regression equations without graphical nomogram visualization, requiring professional statistical calculation and being inconvenient for rapid bedside risk screening by frontline clinicians (23); (2) Insufficient multi-dimensional model validation: A large proportion of published models only complete internal discrimination verification via ROC curves, lacking calibration curve assessment of prediction accuracy and decision curve analysis (DCA) to quantify clinical net benefit, which cannot fully prove clinical practicability; (3) Omission of psychological and social protective/risk factors: Traditional models mainly include physiological and disease indicators such as age, hypertension and stroke type, while ignoring modifiable psychological and social factors (post-stroke depression, cognitive function, self-efficacy, perceived social support) confirmed to strongly affect rehabilitation adherence and functional recovery.

Fourth, existing recurrent stroke-related prediction models mostly focus on predicting stroke recurrence or readmission risk alone, rather than taking post-stroke disability as the primary outcome endpoint. Few studies integrate clinical, psychological and social multi-dimensional predictors to construct a fully validated visual nomogram for disability risk stratification among rehospitalized recurrent stroke patients, resulting in insufficient targeted intervention reference for clinical rehabilitation management.

Collectively, clinical practice is urgently lacking an intuitive, fully validated individualized nomogram prediction tool specially designed for readmitted recurrent stroke patients to screen disability risk. Conventional stroke disability models cannot adapt to the unique clinical characteristics and risk spectrum of this high-disability subgroup, leaving clinicians without quantitative risk stratification evidence to carry out early targeted intervention.

Therefore, this study systematically screens independent risk and protective factors of disability in readmitted patients with recurrent stroke based on multi-dimensional clinical, psychological and social indicators, and establishes a visualized nomogram prediction model comprehensively validated by ROC curve, calibration curve and decision curve analysis. This research aims to fill the above identified research gaps, provide convenient quantitative risk assessment tools for clinical medical staff to identify high disability risk populations of recurrent stroke readmission, and deliver evidence for formulating individualized preventive and rehabilitation intervention strategies to slow functional decline and reduce disability occurrence in such patients.

## Materials and methods

### Study design and participants

From September 2023 to February 2025, a total of 415 recurrent stroke readmitted patients were enrolled from the neurology departments of tertiary hospitals in Liaoning and Shanxi Provinces. Inclusion criteria: ① Diagnosed with stroke in accordance with Chinese Diagnostic Criteria for Major Cerebrovascular Diseases 2019 (24, 25); ② Patients hospitalized due to stroke recurrence; ③ Lesions consistent with clinical manifestations confirmed by cranial CT or MRI examination; ④ Voluntary participation with signed informed consent. Exclusion criteria: ① Diseases impairing motor function, including deep venous thrombosis, fracture, arthritis and amputation; ② Neurodegenerative diseases such as Alzheimer’s disease, epilepsy and amyotrophic lateral sclerosis; ③ Severe hepatic or renal insufficiency, respiratory failure and malignant tumors; ④ Patients with acute mental disorders or coma. The sample size was estimated based on the Logistic regression formula: *n* = number of independent variables ×(5 ∼ 10)÷prevalence rate. Literature reported the stroke disability rate ranged from 38.2 to 62.8% (26). Twenty influencing factors were included, with 5 to 10 cases assigned per variable. Allowing for 20% invalid samples, the minimum required sample size was calculated as 192 cases. Ultimately, 415 eligible subjects were included in this study.

## Methods

### Survey tools

#### General information questionnaire

The questionnaire was self-designed by the research team. It covered gender, age, marital status, educational level, residential area, household income, body mass index, drinking and smoking habits, stroke type, readmission times due to recurrence, complications including hypertension, coronary heart disease, diabetes and hyperlipidemia, as well as regular exercise status.

#### Barthel index (ADL)

Developed by Mahoney and Barthel (27), this scale is the most widely applied assessment tool for basic activities of daily living among stroke patients. It consists of 10 evaluation items including wheelchair transfer, dressing, stair climbing, toileting, bathing, urinary control, feeding and walking, which comprehensively assess the presence and severity of disability. The total score ranges from 0 to 100. Scores ≥95 indicate no disability, while scores below 95 suggest disability. Scores of 60–95 correspond to mild disability, 40–60 to moderate disability, and scores ≤40 to severe disability.

#### Montreal cognitive assessment (MoCA)

The Montreal Cognitive Assessment (MoCA) was developed by Nasreddine et al. (28) in Canada in 2004. Optimized based on the Mini-Mental State Examination, it is applied to evaluate cognitive function of stroke patients. The scale contains 11 items covering 8 cognitive domains, with a total score of 30 points. A total score below 26 points indicates cognitive dysfunction.

#### Self-rating depression scale (SDS)

The scale was compiled by Zung (29) in 1965, containing 20 items divided into 4 dimensions. It adopts 4-point Likert scoring method, with raw scores ranging from 20 to 80. Scores below 50 suggest no depression, and scores of 50 and above indicate depressive symptoms.

#### Self-efficacy scale for chronic diseases (SSC)

Developed by Lorig et al. (30) from Stanford University in 1996, this scale is an internationally recognized tool to assess patients’ self-management confidence in symptom control, role function, emotion regulation and medical communication. It comprises 6 items and 2 dimensions, namely symptom management self-efficacy and general disease management self-efficacy. The total score ranges from 6 to 60, with higher scores representing stronger self-efficacy.

#### Perceived social support scale (PSSS)

Revised by Jiang (31), the scale is widely used in domestic clinical and chronic disease research. It consists of 12 items covering support from family, friends and other sources. Adopting 7-point Likert scoring, the total score ranges from 12 to 84. Higher scores reflect higher level of perceived social support.

#### Data collection

All investigators received standardized training on scale administration to fully master the scoring criteria for the Barthel Index, MoCA, SDS and other assessment tools. They were only permitted to conduct on-site data collection after passing a consistency test on simulated cases with a Kappa coefficient ≥ 0.85. The Barthel Index scores of all completed questionnaires were double-checked by two researchers each day; any case with a total score discrepancy greater than 5 points was reassessed the following day to minimize assessment bias. All disability-related indicators and associated covariates were uniformly assessed within 48 h of patient admission, avoiding periods of acute disease fluctuation. For patients capable of independent communication, the Barthel Index was scored via self-report combined with on-site observation of practical daily activities. For patients with severe aphasia or cognitive impairment, their long-term primary caregivers were asked to describe their routine self-care performance, and assessors adjusted the scores based on direct observation of the patients’ physical mobility. Indicators strongly correlated with disability, including cognitive function, depressive symptoms, physical activity habits, social support and self-efficacy, were measured concurrently. All scales were completed independently with item-by-item scoring to ensure no sub-item scores were omitted. The total Barthel Index score and disability stratification of each paper questionnaire were marked separately. A database was established using double data entry by two independent researchers. Logical validation rules were programmed to automatically flag cases with abnormal total Barthel Index scores, which were manually verified and corrected by referencing the original questionnaires, guaranteeing complete, accurate disability classification data free of logical gaps and entry errors. A total of 419 questionnaires were distributed, and 415 valid ones were retrieved, with an effective recovery rate of 99%.

### Statistical analysis

SPSS 26.0 statistical software was adopted for data analysis with the test level set at 0.05. R Studio was used to establish the nomogram prediction model. ① Descriptive statistics were performed on general information and scale scores. ② Univariate screening: All demographic, clinical, psychological and comorbidity variables were firstly subjected to univariate analysis (chi-square test / independent t-test). Variables with *p* < 0.05 were preliminarily screened out as candidate predictors for multivariate regression. ③ Multivariate regression fitting: We adopted the forward likelihood ratio stepwise regression method to establish the final model. The inclusion criterion for variables was α = 0.05, and the exclusion criterion was α = 0.10. Only independent variables that maintained statistical significance after adjustment of other confounding factors were retained in the final regression equation. ④ The independent variables in the final logistic regression were applied to establish the nomogram via R Studio 4.2.2. The area under ROC curve was used to evaluate discrimination efficiency, calibration curve for predictive accuracy, and decision curve analysis (DCA) for clinical applicability.

## Results

### General information of study participants

A total of 415 recurrent stroke readmitted patients were enrolled in this study, including 228 males and 187 females. In terms of age, 124 patients were under 60 years old, 168 aged 60 to 70 years, and 123 over 70 years. For educational background, 124 patients had primary school education or below, 165 had junior or senior high school education, and 126 held college degree or above. There were 166 non-disabled cases and 249 disabled cases, with a disability incidence rate of 60.0%. Other baseline characteristics are shown in Table 1.

**Table 1:** Univariate analysis of disability in readmitted patients with recurrent stroke (Cases).

Characteristics	Number	No-disability *n* = 166	Disability *n* = 249	χ^2^ /Z	*p*	Estimated effect size
Gender				χ^2^ = 0.026	0.872	0.008
Male	228	92	136			
Female	187	74	113			
Age (years)				χ^2^ = 38.274	<0.001	0.304
<60	124	74	50			
60 ~ 70	168	66	102			
>70	123	26	97			
Marital status				χ^2^ = 1.167	0.280	0.053
No spouse	113	50	63			
Have a spouse	302	116	186			
Education level				χ^2^ = 8.251	0.016	0.141
Primary school and below	124	39	85			
Junior high school/senior high school	165	65	100			
College degree or above	126	62	64			
Place of residence				χ^2^ = 0.583	0.445	0.037
Town	222	85	137			
Rural area	193	81	112			
Economic income				χ^2^ = 2.014	0.365	0.07
<2,000	107	37	70			
2,000 ~ 4,000	186	80	106			
>4,000	122	49	73			
Drinking				χ^2^ = 0.274	0.600	0.026
No	229	89	140			
Yes	186	77	109			
Smoking				χ^2^ = 1.602	0.206	0.062
No	247	105	142			
Yes	168	61	107			
Body mass index (BMI)				χ^2^ = 13.312	0.001	0.179
<18.5	48	28	20			
18.5–23.9	263	109	154			
≥24	104	29	75			
Frequency of incidence				χ^2^ = 39.914	<0.001	0.310
1	200	110	90			
2 ~ 4	180	52	128			
>4	35	4	31			
Stroke type				χ^2^ = 8.437	0.004	0.143
Ischemic stroke	328	143	185			
Hemorrhagic stroke	87	23	64			
Combined hypertension				χ^2^ = 43.445	<0.001	0.324
No	112	74	38			
Yes	303	92	211			
Concomitant heart disease				χ^2^ = 2.310	0.129	0.075
No	327	137	190			
Yes	88	29	59			
Concomitant diabetes				χ^2^ = 10.990	0.001	0.163
No	295	133	162			
Yes	120	33	87			
Hyperlipidemia				χ^2^ = 10.122	0.001	0.156
No	246	114	132			
Yes	169	52	117			
Exercise habit				χ^2^ = 88.231	<0.001	0.461
Often	107	77	30			
Occasionally	226	85	141			
Never	82	4	78			
Depression				χ^2^ = 157.629	<0.001	0.616
No	189	138	51			
Yes	226	28	198			
Cognitive impairment (CI)				χ^2^ = 221.205	<0.001	0.731
No	183	147	36			
Yes	232	19	213			
Self-efficacy(x̄ ± s, points)	415	47.96 ± 6.873	34.11 ± 10.532	t = 14.952	<0.001	0.593
Social support (x ± s, points)	415	61.70 ± 9.726	41.65 ± 9.656	t = 20.665	<0.001	0.713

### Univariate analysis on disability risk of readmitted patients with recurrent stroke

The results of t-test and chi-square test indicated that 13 variables including age, educational level, body mass index, recurrence readmission times, stroke type, complicated diabetes, hypertension, hyperlipidemia, depression, social support, self-efficacy, cognitive status and exercise habit had statistically significant correlations with disability occurrence (*p* < 0.05). No significant differences were observed in other variables (*p* > 0.05), as presented in Table 1.

### Binary logistic regression analysis of disability risk in recurrent stroke readmitted patients

The screened significant variables were taken as independent variables, and disability occurrence was set as the dependent variable for binary Logistic regression analysis. Variable assignment is shown in Table 2. The results revealed that social support (OR = 0.753, 95% CI: 0.674–0.841) and self-efficacy (OR = 0.894, 95% CI: 0.824–0.970) were protective factors against disability. Hypertension complication (OR = 4.230, 95% CI: 1.128–15.865), depression (OR = 8.473, 95% CI: 2.226–32.254), cognitive impairment (OR = 40.982, 95% CI: 8.683–193.425), occasional exercise (OR = 5.122, 95% CI: 1.032–25.431) and sedentary lifestyle (OR = 20.604, 95% CI: 1.370–309.955) were independent risk factors for disability (*p* < 0.05). The regression equation was: Y = 13.595 + 1.442 × Hypertension+2.137 × Depression−0.284 × Social Support−0.112 × Self−efficacy+3.713 × Cognitive status+1.634 × Exercise habit(1) + 3.026 × Exercise habit(2). Relevant data are displayed in Table 3.

**Table 2:** Assignment of independent variables.

Variable	Assignment
Gender	Male = 1, Female = 2
Age	<60 years old = 1, 60 ~ 70 years old = 2, > 70 years old = 3
Marital status	No spouse = 1, Have a spouse = 2
Education level	Primary school and below = 1, Junior high school/senior high school = 2, College degree or above = 3
Place of residence	Town = 1, Rural area = 2
Economic income	<2,000 = 1, 2,000 ~ 4,000 = 2, > 4,000 = 3
Drinking	No = 0, Yes = 1
Smoking	No = 0, Yes = 1
Body mass index (BMI)	<18.5 = 1, 18.5 ~ 23.9 = 2, ≥ 24 = 3
Frequency of incidence	1 = 1, 2 ~ 4 = 2, > 4 = 3
Stroke type	Ischemic stroke = 1, Hemorrhagic stroke = 2
Combined hypertension	No = 0, Yes = 1
Concomitant heart disease	No = 0, Yes = 1
Concomitant diabetes	No = 0, Yes = 1
Hyperlipidemia	No = 0, Yes = 1
Exercise habit	Often = 1, Occasionally = 2, Never = 3
Depression	No = 0, Yes = 1
Cognitive impairment	No = 0, Yes = 1
Self-efficacy	Measured value
Social support	Measured value

**Table 3:** Binary logistic regression analysis of disability in readmitted patients with recurrent stroke.

Variable	B	SE	Wald χ^2^	OR (95% CI)	*p*
Constant	13.595	3.704	13.468	—	<0.001
Combined hypertension	1.442	0.674	4.574	4.230 (1.128–15.865)	0.032
Depression	2.137	0.682	9.815	8.473 (2.226–32.254)	0.002
Social support	−0.284	0.056	25.472	0.753 (0.674–0.841)	<0.001
Self-efficacy	−0.112	0.042	7.249	0.894 (0.824–0.970)	0.007
Exercise habit			6.122		0.047
Exercise habit (1)	1.634	0.818	3.992	5.122 (1.032–25.431)	0.046
Exercise habit (2)	3.026	1.383	4.785	20.604 (1.370–309.955)	0.029
Cognitive impairment (CI)	3.713	0.792	21.995	40.982 (8.683–193.425)	<0.001

### Construction and validation of nomogram prediction model for disability in recurrent stroke readmitted patients

The nomogram prediction model was established based on predictors screened by binary Logistic regression (Figure 1). The C-index was 0.993 (95% CI: 0.988–0.999), consistent with the area under the ROC curve, indicating satisfactory discriminative validity (Figure 2). The sensitivity, specificity and Youden index were 0.956, 0.976 and 1.936 respectively, with the optimal cut-off value of 0.684. The calibration curve fitted well with the ideal curve. The Hosmer-Lemeshow test showed *χ*^2^ =5.380, *p* = 0.716 > 0.05, suggesting favorable model goodness-of-fit (Figure 3). The decision curve presented an upward-right trend, proving fine clinical applicability. The net benefit reached 55.30% at the cut-off value of 0.684 (Figure 4).

**Figure 1:**
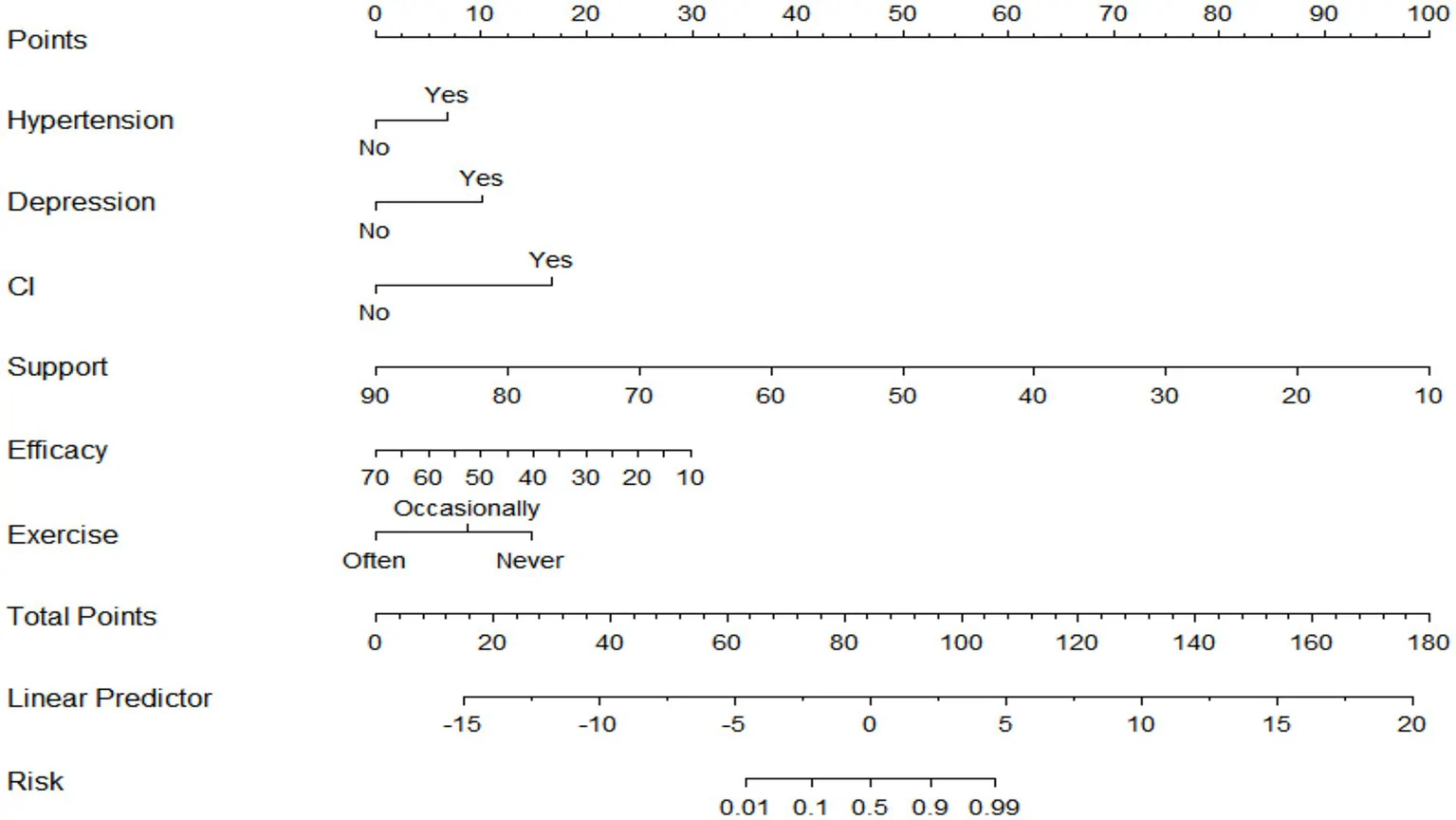
Nomogram model for predicting disability risk in readmitted patients with recurrent stroke.

**Figure 2:**
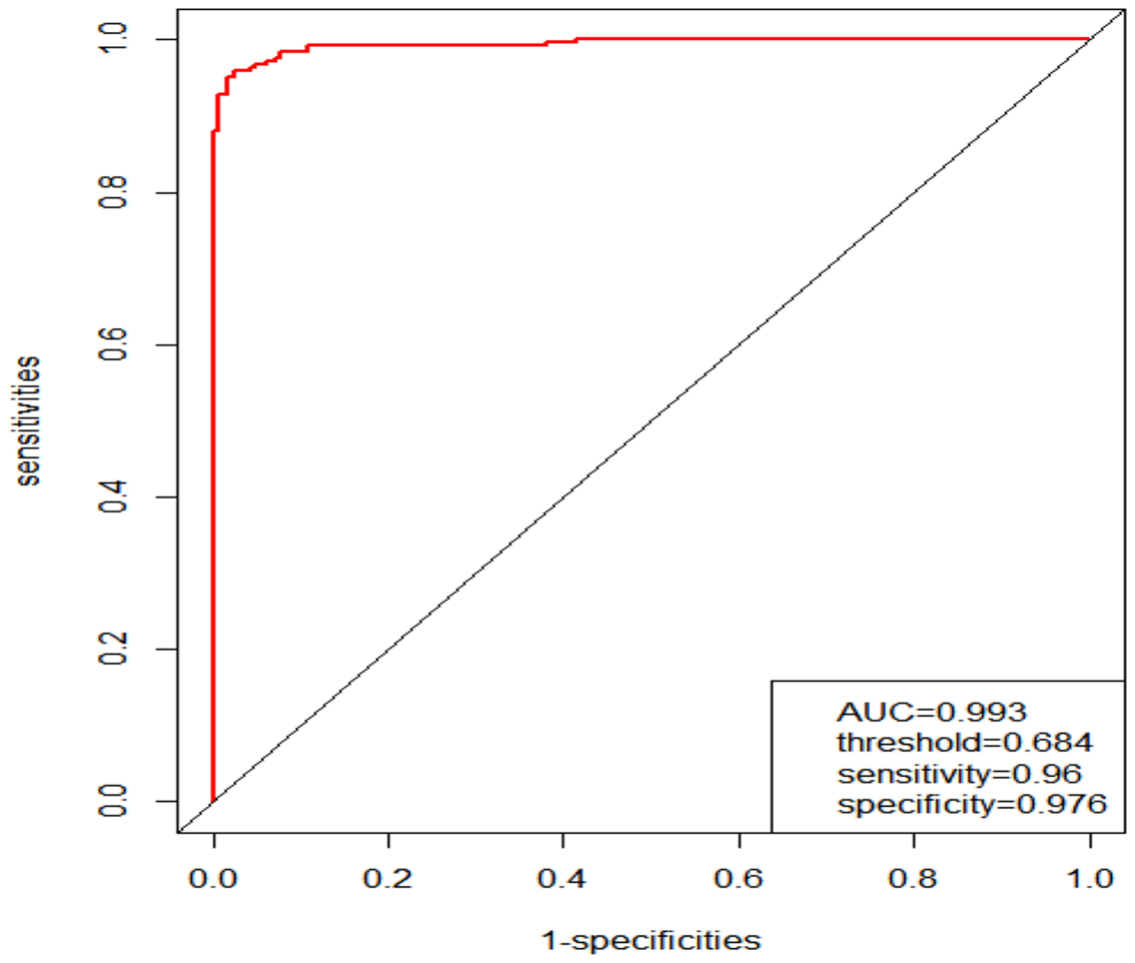
AUC of ROC curve of nomogram model for disability risk prediction.

**Figure 3:**
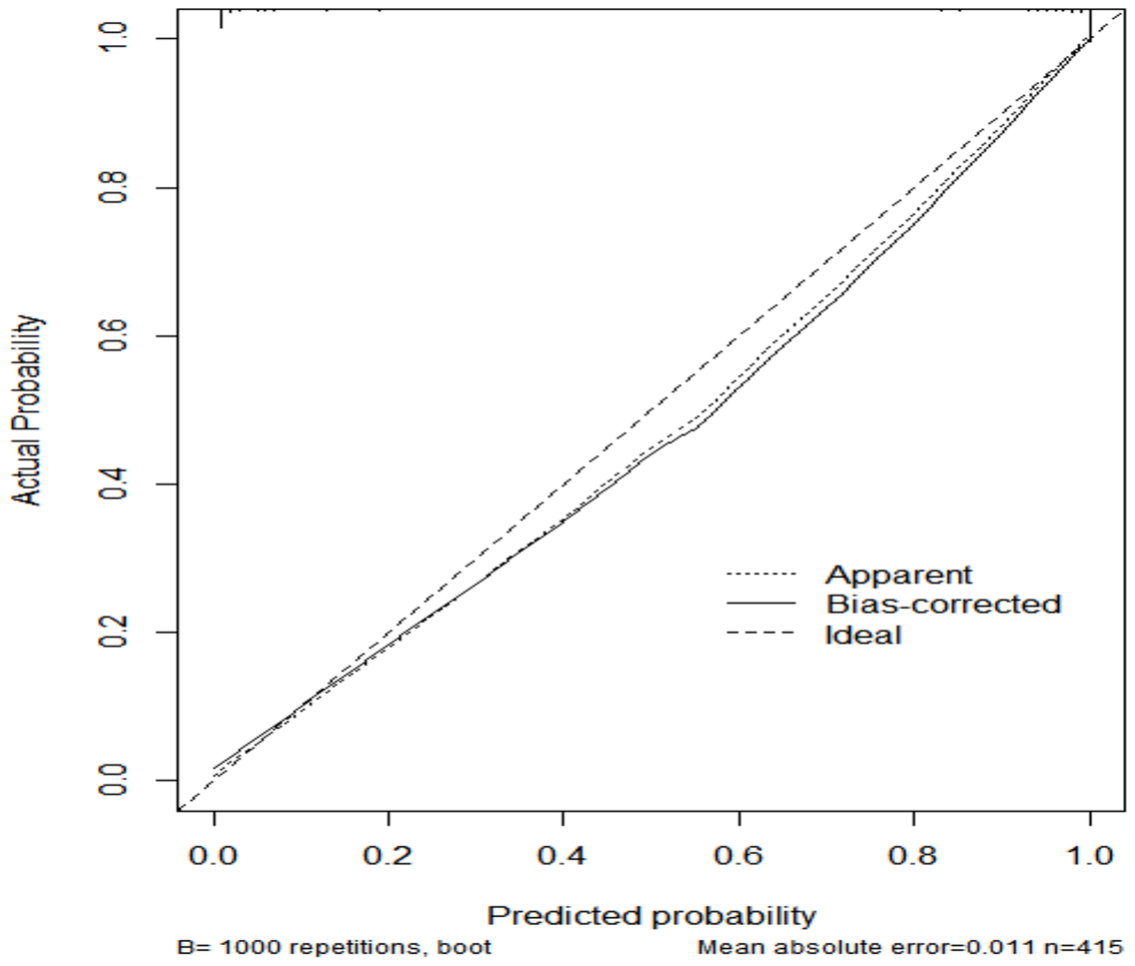
Calibration curve of nomogram model for disability risk prediction.

**Figure 4:**
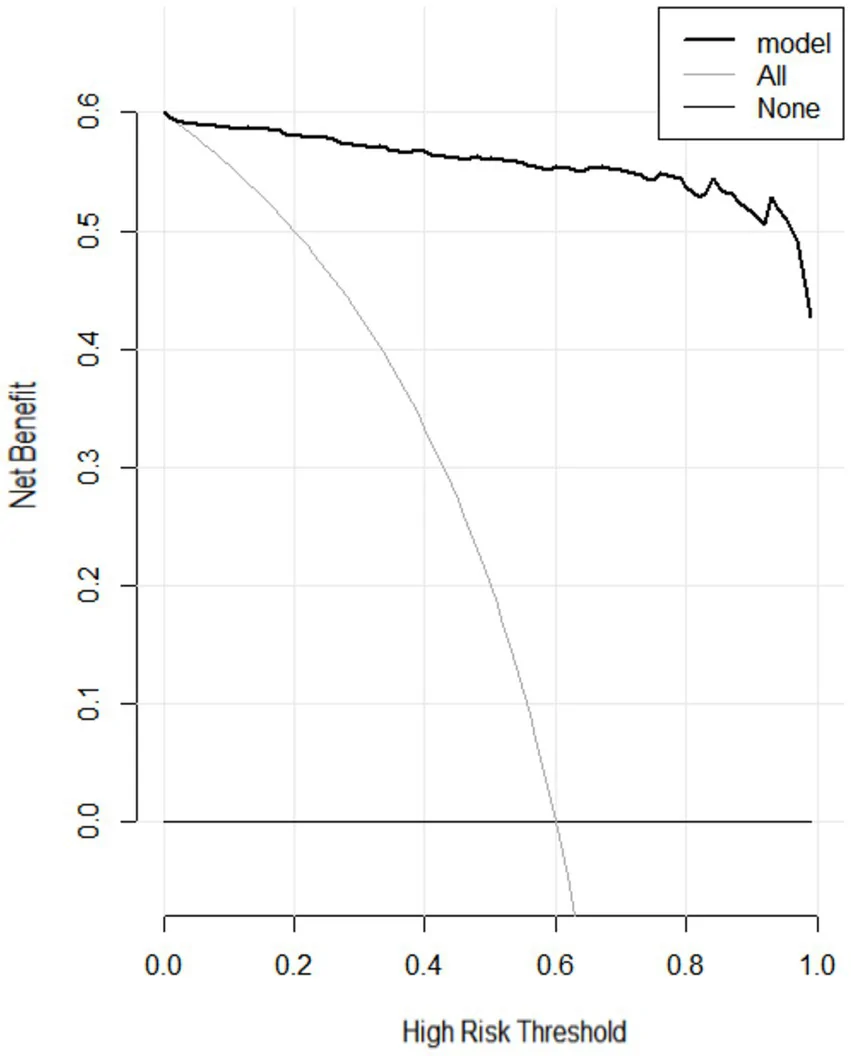
Decision curve analysis of nomogram model for disability risk prediction.

## Discussion

### Analysis of influencing factors of disability in readmitted patients with recurrent stroke

The results indicated that hypertension complication, depression, cognitive impairment, occasional and inactive exercise habits were independent risk factors for disability, while social support and self-efficacy served as protective factors.

1. Independent risk factors with distinct effect characteristics in recurrent readmitted stroke patients.

Hypertension aggravates cerebral vascular endothelial injury, increases cerebral atherosclerosis stenosis and plaque instability, which is a universally accepted risk factor for stroke functional impairment in existing studies (32). However, this study found that hypertension carried a moderate disability risk (OR = 4.230) only in the subgroup of patients with repeated stroke readmission. For first-ever stroke patients, hypertension mainly elevates the risk of initial neurological deficit; while for recurrent rehospitalized patients, long-term cumulative hypertensive vascular damage superimposes repeated cerebral ischemia/hemorrhage lesions, forming a synergistic injury effect. Unlike first-stroke populations whose disability risk mainly originates from single acute brain damage, recurrent readmission patients with hypertension suffer progressive cumulative brain tissue loss, which continuously amplifies the probability of loss of self-care ability after each recurrence. Consistent with Nigerian cohort results of general stroke survivors (33), hypertension predicts worse daily living function, yet this study further clarifies that the adverse effect of hypertension on disability is significantly amplified under the background of repeated stroke attacks and rehospitalization.

Post-stroke depression (PSD) is a well-documented barrier to stroke rehabilitation across existing literature, and this study observed an OR of 8.473 for depression in recurrent readmission patients, far higher than the effect size reported in single-onset stroke cohorts (34). For first-ever stroke patients, depressive symptoms mostly arise from sudden physical dysfunction and short-term psychological shock; in contrast, recurrent stroke patients experience repeated hospitalization, persistent functional decline and long-term care burden, forming chronic, refractory negative emotional states. Sustained depression reduces rehabilitation initiative, disrupts autonomic nervous regulation, and interacts with recurrent cerebral lesions to produce a compound disability-promoting effect (35). This explains why depressive symptoms impose a far stronger adverse impact on self-care capacity in readmitted recurrent stroke patients than in first-onset cases, highlighting that psychological intervention is more urgent for this high-recurrence subgroup.

Cognitive impairment (CI) is confirmed to impair daily living independence in all stroke populations (36), but this study detected an extremely high odds ratio (OR = 40.982) for cognitive dysfunction—the strongest predictor of disability in this cohort, a feature rarely observed in first-ever stroke samples. After single stroke, mild cognitive decline often remains reversible with standardized rehabilitation; recurrent readmission patients suffer repeated multifocal cerebral parenchymal injury, leading to irreversible, progressive cognitive damage covering memory, executive function and motor planning (37, 38). Severe cognitive deficits directly reduce rehabilitation compliance: patients cannot understand training guidance, fail to complete daily self-care independently, and cannot cooperate with secondary prevention management. The wide 95% confidence interval (8.683–193.425) of CI OR is attributed to imbalanced subgroup distribution in this convenience sampling cohort, but the dominant predictive weight of cognitive impairment still reveals a core clinical distinction: cognitive function status becomes the decisive factor determining disability outcomes for patients with repeated stroke hospitalization, rather than a secondary auxiliary predictor as seen in first-stroke groups. Future stratified multi-center research will expand mild cognitive impairment sample size to stabilize effect value estimation.

Sedentary behavior is widely reported to hinder stroke functional recovery (39, 40), yet the gradient risk difference observed in this study represents a population-specific feature of recurrent readmission patients. Relative to patients who maintain regular exercise, occasional exercise elevates disability risk 5.122 times, and complete inactivity raises risk to 20.604 times. For first-ever stroke survivors, interrupted exercise only delays short-term rehabilitation progress; recurrent stroke patients with persistent inactivity face dual damage: insufficient physical training worsens limb motor dysfunction, and poor cardiovascular regulation further increases the risk of stroke relapse, creating a vicious cycle of “inactivity → recurrence readmission → aggravated disability” (41). Long-term sedentary habits formed between multiple hospitalizations continuously offset the rehabilitation gains of previous treatment, which explains why physical inactivity exhibits a graded, exponentially increased disability risk only in the recurrent readmission population.

1. Protective factors: amplified buffering effects of social support and self-efficacy in recurrent stroke readmission subgroups.

Social support and self-efficacy are recognized protective factors for stroke functional recovery in prior research, but their mitigating effects on disability are significantly stronger in patients with repeated stroke hospitalization compared with first-onset patients.

Social support alleviates post-stroke negative emotions and facilitates rehabilitation adherence in general stroke cohorts (42). For recurrent readmission patients, repeated hospitalization brings heavy economic, physical and psychological pressure; adequate family, friend and medical support acts as a critical buffer against the combined adverse effects of hypertension, depression and cognitive impairment. This study verified that each unit increase in social support score reduces disability odds by 24.7%, a larger protective effect size than most single-onset stroke studies. Sufficient social support can help patients persist in regular exercise, accept psychological counseling and complete long-term cognitive training, weakening the synergistic disability-promoting interaction of multiple risk factors—a regulatory mechanism rarely emphasized in research targeting first-ever stroke populations (43).

High self-efficacy strengthens patients’ willingness to participate in rehabilitation and disease self-management. For first-stroke patients, self-efficacy mainly affects short-term rehabilitation enthusiasm; recurrent stroke patients face repeated disease setbacks, which easily induce abandonment psychology (44, 45). High self-efficacy enables patients to actively cope with multiple comorbidities, depressive mood and cognitive obstacles during long-term disease progression, breaking the cycle of recurrence, inactivity and disability (46). The protective OR value of 0.894 in this study proves that self-management confidence is a more critical protective resource for patients with multiple stroke hospitalizations than for single-onset survivors.

### Favorable predictive performance of the nomogram model for disability

Independent influencing factors screened via binary Logistic regression were adopted to establish the nomogram prediction model. ROC curve and calibration curve were applied to verify discrimination and calibration ability, confirming high accuracy and reliability of the model.

Discrimination refers to the capacity to distinguish patients with high and low risks of adverse outcomes, commonly assessed by C-index and AUC (47). An AUC below 0.70 indicates poor discrimination, 0.70–0.90 represents moderate validity, and values over 0.90 suggest excellent discrimination. The AUC of this model was 0.993, demonstrating outstanding discriminatory power. Higher specificity corresponds to lower misdiagnosis rate, while higher sensitivity means lower missed diagnosis rate. With specificity of 0.976 and sensitivity of 0.956, the model presents low risks of misdiagnosis and missed diagnosis.

Calibration reflects the consistency between predicted and actual incidence probabilities (48). Poor calibration leads to underestimated or overestimated risk values, which can be visualized by calibration curves. The ideal curve is a straight line with a slope of 1 passing through the origin. Closer fitting indicates higher predictive accuracy. Both original and corrected calibration curves fitted well with the ideal curve, with a mean absolute error of 0.01, revealing great consistency between predicted and actual risks. In decision curve analysis, the horizontal axis stands for risk threshold and the vertical axis represents net clinical benefit. The net benefit reached 55.30% at the cut-off value of 0.684, proving satisfactory clinical applicability.

Nomogram visually quantifies risks through parallel scales, consisting of score scale, variable indicators and outcome scale. Clinical application procedures are as follows: map each variable value to the score axis and sum up total points; draw a vertical line from total points to obtain the individual disability risk probability. The established nomogram realizes visualized risk assessment, facilitating dynamic disability evaluation for recurrent stroke patients. Targeted interventions based on relevant risk factors can effectively reduce disability incidence and improve patients’ quality of life.

Most existing risk assessment tools for post-stroke disability only adopt single physical indicators such as age, stroke subtype and hypertension, or separate scale scores including MoCA and SDS, which carry prominent clinical limitations. The nomogram model established in this study for readmitted patients with recurrent stroke has three innovative strengths. First, breaking the limitation of traditional predictive tools that merely incorporate demographic and disease-related indicators, this model simultaneously integrates physical comorbidities (hypertension), psychological status (post-stroke depression and cognitive impairment), physical activity habits, as well as two psychosocial protective factors (social support and self-efficacy). It fully depicts the unique compound risk profile of this patient population formed by cumulative repeated cerebral injury, long-term chronic diseases, persistent negative psychological stress and interrupted rehabilitation, which cannot be achieved by single-indicator evaluation systems. Second, this nomogram serves as a visual bedside assessment tool specially developed for the recurrent readmission subgroup with high disability risk. Different from conventional regression models that only generate complex mathematical formulas requiring professional statistical calculation, it converts seven independent predictive factors into simple parallel scoring axes. Clinicians can score each variable and sum the total points upon patient admission, and obtain individualized disability risk probability rapidly without auxiliary software, filling the research gap of specialized visual predictive tools for this patient group. Third, this study completed comprehensive multi-dimensional validation in strict accordance with the TRIPOD reporting guidelines for prognostic models. Unlike most previous models that only conduct internal discrimination validation via ROC curves without calibration testing and clinical benefit evaluation, this research constructed a triple verification system combining the ROC curve with an AUC of 0.993, a well-fitted calibration curve with a mean absolute error of merely 0.01, and decision curve analysis showing a maximum clinical net benefit of 55.30% at the optimal cut-off value of 0.684. It fully verifies the stable predictive performance and prominent clinical practicability of the model, with far superior methodological integrity compared with traditional single-indicator assessment approaches.

In this study, participants were recruited solely from the neurology departments of tertiary hospitals in Liaoning and Shanxi Provinces using convenience sampling, a sampling method with notable limitations. Liaoning is situated in Northeast China and Shanxi in North China. There exist regional disparities between these two provinces and South, Southwest, Central and Northwest China in terms of residents’ lifestyles, the prevention and control of chronic diseases, medical accessibility, the distribution of stroke subtypes, and rehabilitation intervention conditions following recurrent stroke. Consequently, the demographic and clinical characteristics of our sample fail to fully represent the national overall population of readmitted patients with recurrent stroke. Therefore, we plan to conduct multi-center studies in the future to further improve the predictive accuracy and clinical applicability of the model.

## Limitations

This study has several limitations. First, this is a cross-sectional study that only analyzed the immediate effects of clinical, psychological and social factors on disability at the time of patients’ admission. No long-term dynamic follow-up was performed, so the long-term causal relationships and changing trends between each risk factor and disability outcomes could not be clarified. Second, external validation of the nomogram prediction model has not been conducted in the present research. Independent cohort data from multiple regions are required for subsequent external validation to further improve the predictive accuracy and clinical applicability of the model, thereby facilitating its wide application in clinical stroke diagnosis and treatment.

## Conclusion

This study found a relatively high disability incidence among readmitted patients with recurrent stroke. Hypertension complication, depression, cognitive impairment, occasional and sedentary exercise habits were identified as independent risk factors, while social support and self-efficacy served as protective factors. The established prediction model possesses excellent predictive performance and can effectively identify high-risk populations. Medical staff should conduct early assessment based on relevant influencing factors, implement targeted interventions to prevent and reduce disability, facilitate functional recovery and improve patients’ quality of life.

## Data Availability

The raw data supporting the conclusions of this article will be made available by the authors, without undue reservation.

## References

[1] Boursin P Paternotte S Dercy B Sabben C Maïer B. Sémantique, épidémiologie et sémiologie des accidents vasculaires cérébraux [Semantics, epidemiology and semiology of stroke]. Soins. (2018) 63:24–7. doi: 10.1016/j.soin.2018.06.008, 30213310

[2] Dong Y Cao W Cheng X Fang K Zhang X Gu Y . Risk factors and stroke characteristic in patients with postoperative strokes. J Stroke Cerebrovasc Dis (2017);26:1635–1640. doi:10.1016/j.jstrokecerebrovasdis.2016.12.017. 3. 28478979.

[3] Wang LD Peng B Zhang HQ Wang YL Liu M Shan CL . Summary of China stroke prevention and control report 2020. Chin J Cerebrovasc Dis. (2022) 19:136–44.

[4] GBD 2019 Stroke Collaborators. Global, regional, and national burden of stroke and its risk factors, 1990-2019: a systematic analysis for the global burden of disease study 2019. Lancet Neurol. (2021) 20:795–820. doi: 10.1016/s1474-4422(21)00252-0, 34487721 PMC8443449

[5] Wang W Jiang B Sun H Ru X Sun D Wang L . Prevalence, incidence, and mortality of stroke in China: results from a nationwide population-based survey of 480 687 adults. Circulation. (2017) 135:759–71. doi: 10.1161/CIRCULATIONAHA.116.025250 28052979

[6] Li ZX Jiang Y Li H Li Z Xian Y Wang Y. China's response to the rising stroke burden. BMJ. (2019) 364:l879. doi: 10.1136/bmj.l879, 30819725 PMC6394375

[7] Thomas JW, Holloway JJ. Investigating early readmission as an indicator for quality of care studies.Med Care, (1991); 29:377–394, doi: 10.1097/00005650-199104000-00006, 1902276.

[8] Thomgren M, Westling B, Norrving B. Outcome after stroke in patients diseharged to independent living〔J〕. Stroke, (1990); 21: 236–240. 10.1161/01.str.21.2.236 2305398

[9] Markus HS. Reducing disability after stroke. Int J Stroke. (2022) 17:249–50. doi: 10.1177/17474930221080904, 35191348

[10] Burton JK Ferguson EEC Barugh AJ Walesby KE MacLullich AMJ Shenkin SD . Predicting discharge to institutional long-term care after stroke a systematic review and meta analysis. J Am Geriatr Soc. (2018) 66:161–9. doi: 10.1111/jgs.15101, 28991368 PMC5813141

[11] Arba F Ali M Quinn TJ Hankey GJ Lees KR Inzitari D . Lacunar infarcts, depression, and anxiety symptoms one year after stroke. J Stroke Cerebrovasc Dis. (2016) 25:831–4. doi: 10.1016/j.jstrokecerebrovasdis.2015.12.018, 26778600

[12] Lu JH. Zou JH Lü YM Jin RM Chen ZH. Acceptance of disability level and influencing factors in patients with post-stroke disability. Chin Nurs Manag. (2015) 15:796–9.

[13] An YC Chen YX Wang YX Zhao XJ Wang Y Zhang J . Analysis of risk factors for recurrence of ischemic stroke and construction of a prediction model. Chin. J. Epidemiol. (2011) 32:816–20.

[14] Mutai H Furukawa T Araki K Misawa K Hanihara T. Long-term outcome in stroke survivors after discharge from a convalescent rehabilitation ward. Psychiatry Clin Neurosci. (2013) 67:434–40. doi: 10.1111/pcn.12075 23941061

[15] Mohan KM Wolfe CD Rudd AG Heuschmann PU Kolominsky-Rabas PL Grieve AP. Risk and cumulative risk of stroke recurrence: a systematic review and meta-analysis. Stroke. (2011) 42:1489–94. doi: 10.1161/STROKEAHA.110.602615, 21454819

[16] Lin B Zhang Z Mei Y Wang C Xu H Liu L . Cumulative risk of stroke recurrence over the last 10 years: a systematic review and meta-analysis. Neurol Sci. (2021) 42:61–71. doi: 10.1007/s10072-020-04797-5, 33040195

[17] Wang P Wang Y Zhao X Du W Wang A Liu G . In-hospital medical complications associated with stroke recurrence after initial ischemic stroke: a prospective cohort study from the China National Stroke Registry. Medicine (Baltimore). (2016) 95:e4929. doi: 10.1097/MD.0000000000004929, 27631271 PMC5402614

[18] Zhang Y Bai Y Wu XX Zhao YN Liu Y Wang H . Analysis on disability status and influencing factors of readmitted patients with recurrent ischemic stroke. Chinese General Practice Nursing. (2021) 19:4287–90.

[19] Abdulaziz KE Perry JJ Yadav K Dowlatshahi D Stiell IG Wells GA . Quality and transparency of reporting derivation and validation prognostic studies of recurrent stroke in patients with TIA and minor stroke: a systematic review. Diagn Progn Res. (2022) 6:9. doi: 10.1186/s41512-022-00123-z PMC9118704 35585563

[20] Cheng S Wei Y Zheng H Zhang Q Yang X Lu Z . Artifical intelligence-powered delta-NIHSS-based model for predicting recurrence, disability and mortality after acute ischaemic strokes (DISCO): a modelling study. EClinicalMedicine. (2025) 90:103684. doi: 10.1016/j.eclinm.2025.103684 PMC12753274 41477565

[21] Ma Y Jin Z Yi X Ye X. Risk factors analysis and nomograph model construction of unplanned readmission for ischemic stroke within 31 days in Wenzhou. Front Neurol. (2025) 16:1499564. doi: 10.3389/fneur.2025.1499564 PMC11983885 40212613

[22] Hua Y Yan C Zhou C Zheng Q Li D Tu P. Risk prediction models for intracranial hemorrhage in acute ischemic stroke patients receiving intravenous alteplase treatment: a systematic review. Front Neurol. (2024) 14:1224658. doi: 10.3389/fneur.2023.1224658, 38249727 PMC10799340

[23] Jin J Ma P Wang E Xie Y. Risk factors of recurrence of acute ischemic stroke and construction of a nomogram model for predicting the recurrence risk based on lasso regression. Nan Fang Yi Ke Da Xue Xue Bao. (2024) 44:2375–81. doi: 10.12122/j.issn.1673-4254.2024.12.13 PMC11683344 39725626

[24] Chinese Society of Neurology, Cerebrovascular Disease Group of Chinese Society of Neurology. Diagnostic criteria for major cerebrovascular diseases in China, 2019. Chin J Neurol. (2019) 52:710–5. doi: 10.1007/978-1-59745-078-2_2

[25] Moons KG Altman DG Reitsma JB Moons KGM Ioannidis JPA Macaskill P . Transparent reporting ofa multivariable prediction mode for individual prognosis or diagnosis (TRIPOD): explanation and elaboration. Ann Intern Med. (2015) 162:W1–W73. doi: 10.7326/m14-0698, 25560730

[26] Li NR Liu LL Huang Y Qin LH. Research status of long-term care models for disabled stroke patients. Chin Nurs Res. (2020) 34:1566–8.

[27] Eriksson M Norrving B Terént A Stegmayr B. Functional outcome 3 months after stroke predicts long-term survival. Cerebrovasc Dis. (2008) 25:423–9. doi: 10.1159/000121343, 18349536

[28] Nasreddine ZS Phillips NA Bédirian V Charbonneau S Whitehead V Collin I . The Montreal cognitive assessment, MoCA: a brief screening tool for mild cognitive impairment. J Am Geriatr Soc. (2005) 53:695–9. doi: 10.1111/j.1532-5415.2005.53221.x, 15817019

[29] Zung WW. A self-rating depression scale. Arch Gen Psychiatry. (1965) 12:63–70. doi: 10.1001/archpsyc.1965.01720310065008, 14221692

[30] Lorig KR Sobel DS Ritter PL Laurent D Hobbs M. Effect of a self-management program on patients with chronic disease. Eff Clin Pract. (2001) 4:256–62. 11769298

[31] Jiang QJ. Medical Psychology. Beijing: People’s Medical Publishing House (2004). p. 82–4.

[32] Xu L Liu Y He H Fields NL Ivey DL Kan C. Caregiving intensity and caregiver burden among caregivers of people with dementia: the moderating roles of social support. Arch Gerontol Geriatr, (2021), 94:104334. doi: 10.1016/j.archger.2020.104334 33516077

[33] Oyewole OO Ogunlana MO Oritogun KS Gbiri CA. Post-stroke disability and its predictors among Nigerian stroke survivors. Disabil Health J. (2016) 9:616–23. doi: 10.1016/j.dhjo.2016.05.011, 27387815

[34] Frank D Gruenbaum BF Zlotnik A Semyonov M Frenkel A Boyko M. Pathophysiology and current drug treatments for post-stroke depression: a review. Int J Mol Sci. (2022) 23:15114. doi: 10.3390/ijms232315114, 36499434 PMC9738261

[35] Srivastava A Taly AB Gupta A Murali T. Post-stroke depression: prevalence and relationship with disability in chronic stroke survivors. Ann Indian Acad Neurol. (2010) 13:123–7. doi: 10.4103/0972-2327.64643, 20814496 PMC2924510

[36] Astuti P Kusnanto K Dwi Novitasari F. Depression and functional disability in stroke patients. J Public Health Res. (2020) 9:1835. doi: 10.4081/jphr.2020.1835, 32728574 PMC7376455

[37] Jokinen H Melkas S Ylikoski R Pohjasvaara T Kaste M Erkinjuntti T . Post- stroke cognitive impairments common even after successful clinical recovery. Eur J Neurol. (2015) 22:1288–94. doi: 10.1111/ene.12743 26040251

[38] Appelros P Karlsson GM Seiger A Nydevik I. Neglect and anosognosia after first-ever stroke: incidence and relationship to disability. J Rehabil Med. (2002) 34:215–20. doi: 10.1080/165019702760279206, 12392236

[39] Nguyen LTK Do BN Vu DN Pham KM Vu MT Nguyen HC . Physical activity and diet quality modify the association between comorbidity and disability among stroke patients. Nutrients. (2021) 13:1641. doi: 10.3390/nu13051641, 34068135 PMC8152968

[40] Ezeugwu VE Manns PJ. Sleep duration, sedentary behavior, physical activity, and quality of life after inpatient stroke rehabilitation. J Stroke Cerebrovasc Dis. (2017) 26:2004–12. doi: 10.1016/j.jstrokecerebrovasdis.2017.06.009, 28669653

[41] Peçanha T Goessler KF Roschel H Gualano B. Social isolation during the COVID-19 pandemic can increase physical in activity and the global burden of cardiovascular disease. Am J Physiol Circ Physiol. (2020) 318:H1441–6. doi: 10.1152/ajpheart.00268.2020, 32412779 PMC7303725

[42] Sharrief AZ Sánchez BN Lisabeth LD Skolarus LE Zahuranec DB Baek J . The impact of pre-stroke depressive symptoms, fatalism, and social support on disability after stroke. J Stroke Cerebrovasc Dis. (2017) 26:2686–91. doi: 10.1016/j.jstrokecerebrovasdis.2017.06.039, 28774795 PMC5624842

[43] Tramonti F Fanciullacci C Giunti G Rossi B Chisari C. Functional status and qualityoflife of stroke survivors undergoing rehabilitation pro-grammesin a hospital setting. NeuroRehabil. (2014) 35:1–7. doi: 10.3233/nre-141092 24990001

[44] Du BH Ma LH Wang RQ Wang YQ Cui Y Dong X. Effects of motivational interviewing combined with task-oriented training on upper limb function and self-efficacy in stroke patients. Chinese Evidence-Based Nursing. (2022) 8:2489–93.

[45] Vincent-Onabajo GT Lawan AK Oyeyemi AY Hamzat TK. Functional self-efficacy and its determinants in Nigerian stroke survivors. Top Stroke. (2012) 19:411–6. doi: 10.1310/tsr1905-411 22982828

[46] Schmid AA Van Puymbroeck M Altenburger PA Dierks TA Miller KK Damush TM . Balance and balance self-efficacy are associated with activity and participation af- ter stroke:a cross-sectional study in people with chronic stroke. Arch Phys Med Rehabil. (2012) 93:1101–7. doi: 10.1016/j.apmr.2012.01.020, 22502804

[47] Saleh SN Makam AN Halm EA Nguyen OK. Can we predict early 7-day readmissions using a standard 30-day hospital readmission risk prediction model? BMC Med Inform Decis Mak. (2020) 20:227. doi: 10.1186/s12911-020-01248-1, 32933505 PMC7493907

[48] Lu JY. Influencing Factors of Mild Cognitive Impairment and Construction of Risk Prediction Model in Elderly Hypertensive Patients in Community. Nanning: Guangxi University of Chinese Medicine (2021).

